# Short-term treatment with taurolidine is associated with liver injury

**DOI:** 10.1186/s40360-017-0168-z

**Published:** 2017-08-11

**Authors:** René Fahrner, Anika Möller, Adrian T. Press, Andreas Kortgen, Michael Kiehntopf, Falk Rauchfuss, Utz Settmacher, Alexander S. Mosig

**Affiliations:** 1 Department of General, Visceral and Vascular Surgery, University Hospital Jena, 07747 Jena, Germany; 2 Center for Sepsis Control and Care (CSCC), University Hospital Jena, 07747 Jena, Germany; 3 Department of Anesthesiology and Intensive Care Therapy, University Hospital Jena, 07747 Jena, Germany; 4 Department of Clinical Chemistry and Laboratory Diagnostics, University Hospital Jena, 07747 Jena, Germany

**Keywords:** Cytokines, Liver injury, Liver biochip, Taurolidine, Liver enzymes

## Abstract

**Background:**

Taurolidine has been used for peritonitis, oncological and catheter-lock treatment because of its anti-inflammatory properties. It has been suggested that taurolidine has no severe side-effects, but after long-term use morphological and functional changes of the liver were reported. The aim of this study was to investigate the effect of short-term use of taurolidine on the liver.

**Methods:**

In HepaRG cell cultures and on a novel liver biochip dose-dependent effects of taurolidine treatment on hepatocyte adherence and cell viability was investigated. Furthermore, liver enzymes and interleukin- (IL-) 6 were measured in supernatants. Male rats were treated with low- or high-dose taurolidine, respectively, and compared to controls with physiological saline solution administration regarding blood serum parameters and histology.

**Results:**

In HepaRG cell cultures, hepatocyte adherence was significantly decreased, cell death and cleaved caspase-3 were significantly increased after administration of taurolidine in a dose-dependent manner. High-dose application of taurolidine led to elevated liver enzymes and IL-6 secretion in hepatic organoid. After 24 h a significant increase of serum GLDH and ASAT was observed in rats treated with high-dose taurolidine treatment.

**Conclusions:**

Our results suggest that taurolidine caused liver injury after short-term use in in vitro and in vivo models probably due to direct toxic effects on hepatocytes. Therefore, the taurolidine dose should be titrated in further investigations regarding liver injury and inflammation.

**Electronic supplementary material:**

The online version of this article (doi:10.1186/s40360-017-0168-z) contains supplementary material, which is available to authorized users.

## Background

Taurolidine was first introduced decades ago as a broad range antibiotic drug for the treatment of peritonitis, sepsis and pleural empyema [1–4]. Taurolidine is a chemical dimeric molecule of two taurinamide rings [5] which is stable with a half-life time of approximately eight hours [6]. The main anti-inflammatory mechanism results in a reduction of pro-inflammatory cytokine release such as tumor necrosis factor (TNF), interleukin (IL-) 6, and IL-8 [7–13]. In addition, an inactivation of endotoxins was reported [3], as well as anti-microbial, anti-adherent, anti-inflammatory and anti-neoplastic effects [5]. Therefore, taurolidine was used in inflammatory experimental and clinical studies as prophylaxis against catheter associated infections [14–17]. Additionally, taurolidine was investigated in experimental oncology and showed promising results in tumor therapy [18–27]. Several mechanisms were discussed, such as direct apoptosis of the tumor cells [5] or indirectly via inhibition of NF-kappaB and consecutive inhibition of anti-apoptotic regulator leading to cell death [21].

So far, taurolidine was reported to be a well-tolerated drug without relevant side effects in animal studies and in human local peritoneal and pleural application [11, 14, 15, 17, 28, 29]. However, one recent publication reported that long-term high-dose treatment with taurolidine caused severe hepatic injury in a murine osteosarcoma model [30]. Therefore, the authors suggested caution before widespread use of taurolidine as an anti-cancer drug in clinical oncology [31].

Because of the reported anti-inflammatory properties, taurolidine could be useful as a potential drug for acute liver injury and prevention of hepatic ischemia-reperfusion injury in the future. So far toxic effects of taurolidine on liver function and morphology during short-term treatment have not been investigated. Thus, the aim of this pilot study was to evaluate the effect of taurolidine on hepatocytes in vitro using a conventional cell culture setting as well as a novel human liver biochip model. In addition, the short-term effect in the liver after a systemic use of taurolidine was investigated in an experimental rat model.

## Methods

All research protocols were carried out in accordance with the National Institutes of Health guidelines for the care and use of experimental animals and approval of the Animal Care Committee of Thuringia, Germany (No. 22-2684-04-02-061/14).

### Animal preparation and experimental setting

Experiments were performed using male Lewis rats kept at the Central Animal Facility of the Friedrich Schiller University Jena, Germany and were purchased from Charles River, Sulzfeld, Germany. The animals were kept with a 12 h light/dark cycle at 22 °C. All procedures were performed in animals aged 8–10 weeks with a mean weight of 286 ± 28 g. Taurolidine was obtained as a ready-to-use solution with a concentration of 2% (Geistlich Pharma AG, Switzerland). The animals were divided into three groups of six animals each: low dose taurolidine (140 mg/kg body weight), high dose taurolidine (290 mg/kg body weight) and controls with equivalent volume of physiologic saline solution administered intraperitoneally. The harvesting of blood and tissue was performed 24 h after the treatment under general anesthesia with isoflurane inhalation. During general anesthesia laparotomy was performed and a lethal amount of blood was taken from the inferior vena cava. Immediately after removal of blood the liver was excised and processed.

### Immunohistochemical analysis of the liver

Fresh liver tissue was fixed overnight in 4% paraformaldehyde and embedded in paraffin. For hematoxylin/eosin (HE) staining, sections were deparaffinized with xylol and then counterstained with HE. The analysis of all histology slides was performed blinded by the same investigator.

### Cell culture

HepaRG hepatocyte cells were obtained from Biopredic International (Rennes, France). Cells were initially seeded at a density of 2.7 × 10^4^ cells/cm^2^ and cultured in complemented William’s Medium E (Biochrom, Berlin, Germany) containing 10% (*v*/v) FCS (Life Sciences, Darmstadt, Germany), 2 mM glutamine (GIBCO, Darmstadt, Germany), 5 μg/ml insulin (Sigma-Aldrich, Steinheim, Germany), 50 μM hydrocortisone-hemisuccinate (Sigma-Aldrich, Steinheim, Germany) and 100 U/ml penicillin/100 μg/ml streptomycin mixture (GIBCO). In a humidified cell incubator at 5% CO_2_ and 37 °C cells were cultured to grow for 14 days with renewal of the medium every three to four days. As described previously, cell differentiation was induced by addition of 2% DMSO for 14 days. Differentiated cells were used for up to four weeks [32]. Endothelial cells (HUVECs) were isolated from human umbilical cord veins as previously described [33]. Donors gave written consent after they were informed about the aim of the study. HUVEC cells were cultured in Endothelial Cell Medium (ECM) (Promocell, Heidelberg, Germany) up to passage four. Peripheral blood mononuclear cells (PBMCs) were isolated using a Ficoll density gradient centrifugation as previously described [34] and seeded in X-VIVO 15 medium (Lonza, Cologne, Germany) with 10% (*v*/v) autologous human serum, 10 ng/ml human granulocyte macrophage colony-stimulating factor (GM-CSF) (PeproTech, Hamburg, Germany) and penicillin (100 u/ml)/streptomycin (100 μg/ml). After three hours of incubation in a humidified cell incubator at 5% CO_2_ and 37 °C the cells were washed with X-VIVO 15 medium. For 24 h adherent monocytes were cultivated in X-VIVO 15 medium and seeded into liver biochip. LX-2 stellate cells (kindly provided by Scott L. Friedman, Division of Liver Diseases, Mount Sinai School of Medicine, New York City, NY, USA) were cultured in Dulbecco’s Minimum Essential Medium (DMEM) (Biochrom, Berlin, Germany) with supplementation of 10% (*v*/v) FCS, 1 mM sodium pyruvate (GIBCO) and penicillin/streptomycin.

### WST-assay

WST-assay (Roche Diagnostics GmbH) was used to assess cell viability and toxic effects of taurolidine 24 h after HepaRG treatment. 3 × 10^5^/cm^2^ HepaRG cells were cultured in complemented William’s Medium E with or without taurolidine with pre-determined concentrations for 24 h at 5% CO_2_ and 37 °C. Subsequently WST-assay dye was added. Briefly, the stable tetrazolium salt WST-1 is cleaved to formazan, a soluble metabolite, by a complex bioreductive mechanism in viable cells. The amount of formazan dye reflects the number of metabolically viable cells. Therefore, the cell medium and WST dye were prepared according to the manufacturer’s instructions in a 96-well tissue culture plate. Cells were incubated 30 min at 37 °C and 5% CO2 with the WST-reagent. Absorbance was measured at a wavelength of 450 nm with a reference wavelength of 650 nm with an ELISA-Reader, reflecting the number of viable cells.

### Cleaved caspase-3

Liver cells were seeded on 13 mm glass coverslips (Menzel, Braunschweig, Germany) in 24 well plates. After washing twice with DPBS, cells were fixed using a 4% paraformaldehyd solution. After washing with PBS and permeabilisation a 0.1% saponin solution was added for one hour to block the cells. After incubation with the primary antibody against cleaved caspase 3 (Cell Signaling Technology, Leiden, Netherlands) overnight in a humidity chamber in the fridge (at 8 °C), an AF488-flourescence-marked secondary goat anti rabbit antibody (Life Technologies, Karlsruhe, Germany), combined with DAPI (Life Technologies) staining of the cell nucleus was added for 30 min. Microscopy was performed using an Axio Observer Z1 fluorescence microscope with ApoTome.2 equipment (Carl Zeiss AG, Jena, Germany). Finally, the mean fluorescent intensity of at least five samples of controls and high-dose taurolidine each were measured using a computer based program (ImageJ®).

### Biochips

MOTiF biochips, a cyclic olefin copolymer (COC), were made from Topas and obtained from the microfluidic ChipShop GmbH (Jena, Germany). The biochips were manufactured as previously described [35]. Briefly, chips were made by injection molding with integration of a 12 μm thick PET membrane with a pore diameter of 8 μm and a density of 2 × 10^5^ pores/cm^2^ (TRAKETCH Sabeu, Radeberg, Germany). Sealed top and bottom surfaces of the chips and channel structures were obtained by an application of an extruded 140 μm thick COC film using a low-temperature proprietary bonding method. For perfusion and oxygen equilibration during experimentation gas permeable silicon tubing was used. To avoid unfavorable flow conditions and trapping of stationary bubbles the ramping structures have been introduced into the chip bulk. Oxygen plasma treatment for hydrophilization of the whole chip surface reduced bubble formation in the chips. Before use, the perfusion medium was stirred and equilibrated overnight under perfusion conditions. After seeding the cells into the upper and lower biochip chamber, taurolidine was added for stimulation at different doses. After 24 h, supernatants were collected for further analysis.

### Measurement of extracellular liver enzymes in serum and cell culture supernatants

Levels of aspartate aminotransferase (ASAT) and glutamate dehydrogenase (GLDH) were measured in serum and cell culture supernatants using the Abbott Architect ci8200 Integrated System (Abbott Laboratories, Abbott Park, IL, USA) according to the manufacturer’s protocol.

### Measurements of cytokines

Rat serum samples were analyzed for IL-6, IL-1ß, IL-10 and tumor necrosis factor (TNF) alpha levels by enzyme-linked immunosorbent assays using immunoassay kits (Quantikine, R&D System) according to the manufacturer’s protocol. Biochip supernatants were collected at pre-determined time points and immediately frozen at −80 °C. Cytokines (IL-1ß, IL-6, IL-10 and TNF-α) were measured using Cytometric Bead Array (CBA) assay (BD Biosciences) according to the manufacturer’s protocol using standard CBA flex sets. Cytokine analysis was performed on a BD FACS-Canto II cytometer with FACSDiva software with data analysis using FCAP Array V3 software (Softflow, Pecs, Hungary).

### Statistics

All data are expressed as arithmetic means ± standard deviations. For statistical analysis one-way ANOVA test was performed. The statistical analysis was performed with GraphPad Prism Version 5.0 (GraphPad Software, Inc., La Jolla, USA). Statistical differences with *p* < 0.05 were considered as statistically significant.

## Results

### Taurolidine caused disruption of hepatic cell adherence and cell death in vitro

**Fig. 1:**
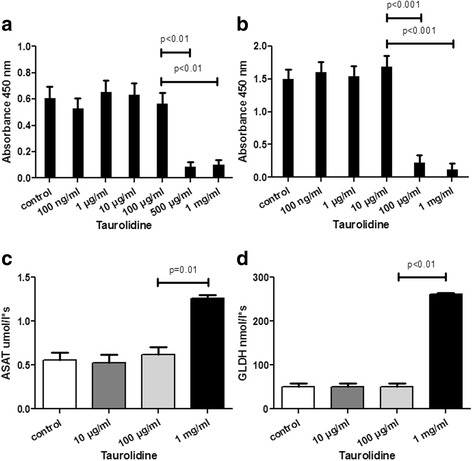
Cell survival of HepaRG cells with taurolidine treatment for 24 h was assessed by WST assay. Cell survival of seeded and adherent HepaRG cells was significantly decreased after treatment with taurolidine concentration of 500 μg/ml (**a**). HepaRG cells without previous adherence showed cell death at a concentration of 100 μg/ml (**b**). Liver enzymes in supernatants of cell cultures were analyzed and showed a significant elevation of ASAT and GLDH with increasing taurolidine concentrations (**c, d**). Representative data of three independent experiments are shown

**Fig. 2:**
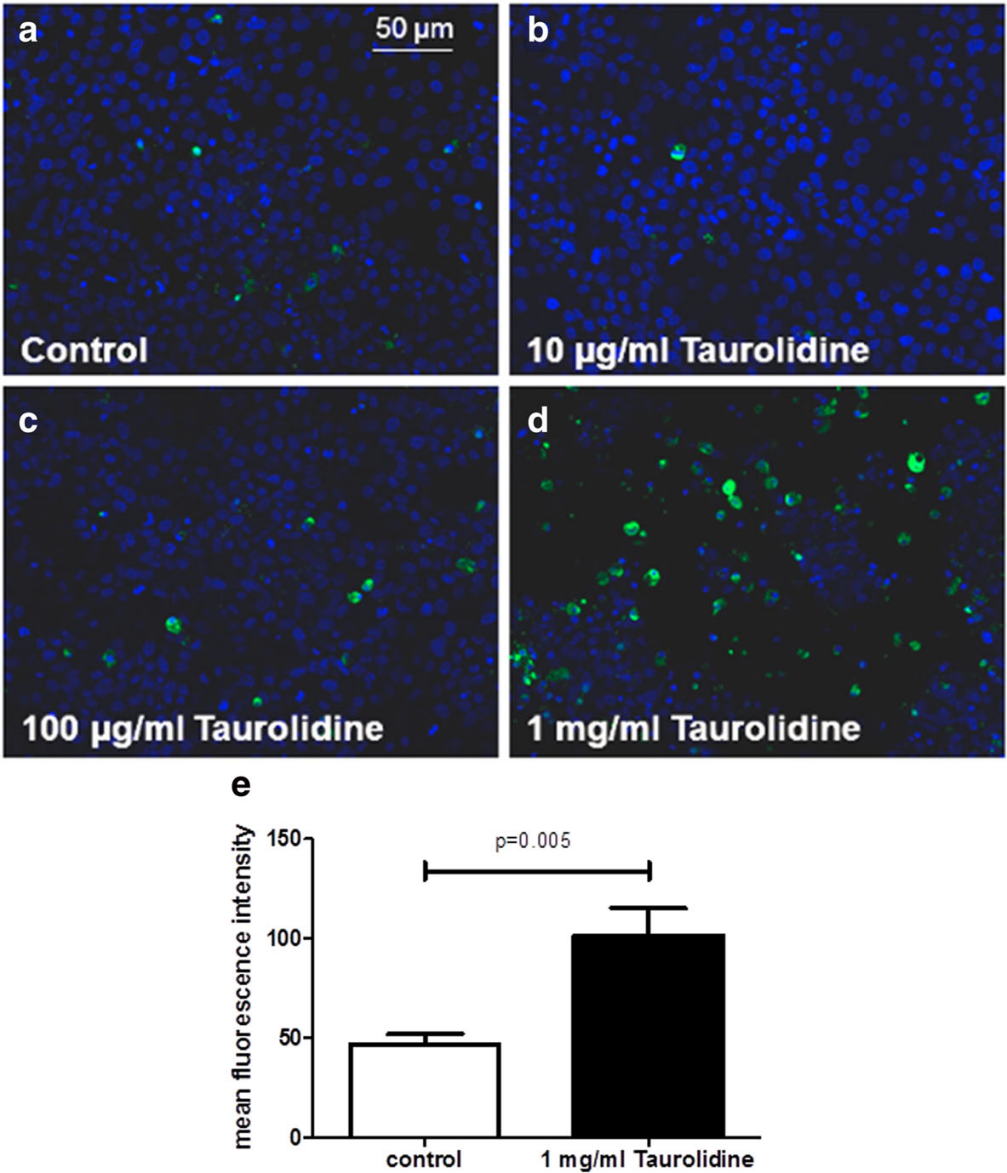
For cell viability investigations previously seeded hepatocytes were treated with taurolidine and stained for cleaved caspase 3. Fluorescence microscopy revealed an increase of cleaved caspase 3 positive cells (*green cells*) in a dose-dependent manner (**a**-**d**). Analysis of mean fluorescent intensity using a computer based program (ImageJ®) showed significantly higher fluorescence after administration of high dose taurolidine (1 mg/ml) (**e**). Representative data of three independent experiments are shown

HepaRG cells were treated with increasing doses of taurolidine in cell cultures. Cell adherence and cell morphology were altered by increasing doses of taurolidine (data not shown). Treatment with taurolidine for 24 h affected HepaRG cell viability with a LC50 of 125 μg/ml (Fig. 1a and Additional file 1: Figure S1). Treatment of hepatocytes with taurolidine without previous surface adherence even showed cell death at a concentration of 100 μg/ml (Fig. 1b), reflecting a higher toxicity on cells without adherence and without direct cell-to-cell contact. Further, the release of liver enzymes in supernatants of cell cultures such as ASAT and GLDH significantly correlated with increasing taurolidine concentrations (Fig. 1c and d). In addition, levels of cleaved caspase 3, a marker for apoptotic cells, were significantly elevated in hepatocytes after taurolidine application (Fig. 2a-e).

### Dose-dependent effect of taurolidine treatment in hepatic biochip

**Fig. 3:**
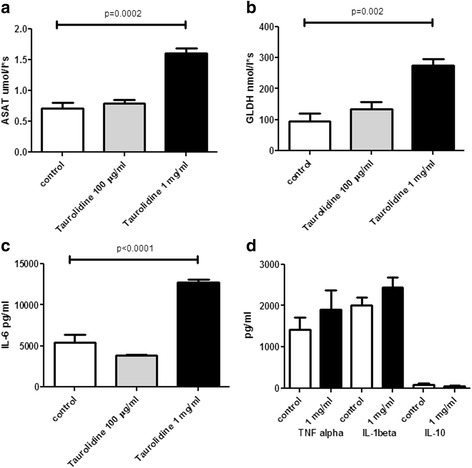
Liver biochips were perfused with taurolidine at a concentration of 100 μg/ml and revealed no significant hepatocyte damage assessed by measurement of liver enzymes in biochip supernatants. Whereas a concentration of 1 mg/ml taurolidine revealed significantly increased levels of ASAT and GLDH (**a**, **b**). IL-6 levels showed similar results, with a significant increase after high-dose treatment (**c**). Serum levels of TNF-α, IL-1ß and IL-10 were not significantly different between controls and taurolidine with 1 mg/ml (**d**). Representative data of three independent experiments are shown

On the basis of the results of HepaRG cell culture experiments with adherent hepatocytes, liver biochips were perfused with a concentration of 100 μg/ml taurolidine, a concentration below the toxic threshold dose in WST assays. Similar to the findings of cell culture experiments, low concentrations of taurolidine caused no significant hepatocyte damage as assessed by measuring liver enzymes in biochip supernatants. A concentration of 1 mg/ml taurolidine however revealed significantly elevated serum levels of GLDH and ASAT (Fig. 3a and b). IL-6 levels were not elevated after perfusion of the liver biochip with low taurolidine concentration but were significantly elevated with high taurolidine concentration (Fig. 3c). There were no statistically significant differences regarding TNF-α, IL-1ß or IL-10 serum levels in the controls compared to high dose taurolidine group (Fig. 3d). Perfusion of the liver biochip with 1 mg/ml taurolidine showed hepatocellular excretory dysfunction of MRP-2 dependent release of 5(6)-Carboxy-2′,7′-dichlorofluorescein (CDF) secretion assessed by fluorescence microscopy (data not shown).

### Short-term treatment with taurolidine without histological alterations

**Fig. 4:**
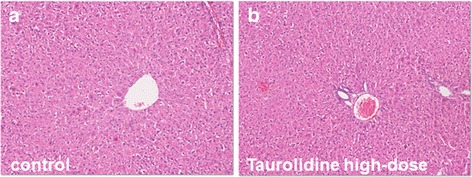
Representative histological figures with H&E staining of rat livers after injection of saline (*n* = 6) or taurolidine (*n* = 6). There were no signs of inflammation or necrosis 24 h after treatment with control (**a**) or high-dose of taurolidine (**b**)

After 24 h post injection of taurolidine or saline no structural and morphological alterations were seen in rat livers (Fig. 4a and b). Treatment with taurolidine caused neither influx of inflammatory cells nor cell ballooning or necrosis.

### Alteration of liver function in vivo

**Fig. 5:**
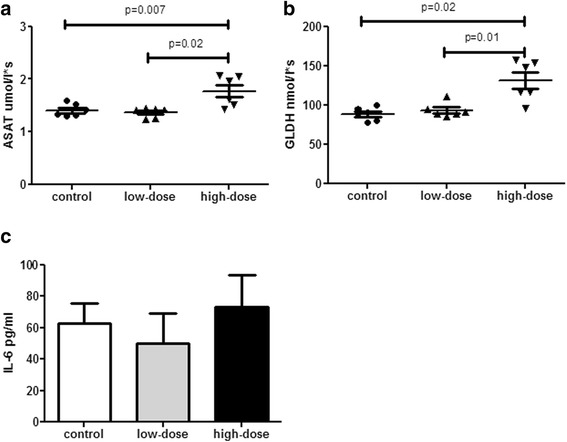
Serum levels of ASAT (**a**), GLDH (**b**) and IL-6 (**c**) in rats 24 h after injection of saline or taurolidine, showing a significant elevation of ASAT and GLDH levels in high-dose taurolidine in comparison to controls or low-dose treatment. Animals per group *n* = 6

Serum levels of ASAT and GLDH in rats were significantly elevated after a single systemic taurolidine treatment with high-dose compared to low-dose taurolidine or controls, whereas there were no differences between the low-dose group and controls (Fig. 5a and b). Serum IL-6 levels 24 h after treatment showed no significant differences between the groups (Fig. 5c).

## Discussion

To the best of our knowledge, this study investigated for the first time the effect of systemic short-term use of taurolidine on liver function and morphology using in vitro models and an in vivo model in rats. In all investigated models the treatment with taurolidine caused liver injury at a specific toxic dose.

During acute liver injury e.g. after liver resection [36, 37], ischemia-reperfusion injury [38], sepsis [39], and acute inflammation [40] a pro-inflammatory cytokine release may occur and is associated with an activation of immune cells, such as Kupffer cells, natural killer (NK) cells, NK-T cells and dendritic cells leading to further liver injury [41–43]. In case of severe liver injury, these effects are associated with consecutive liver failure and death due to multiple organ failure [44, 45]. To date there is no specific treatment of liver injury to prevent organ failure and poor outcome. Therefore, new treatments and drugs are needed to address this problem and taurolidine might be a potential treatment in the future due to its reported impressive anti-inflammatory effects. Hence, this study was planned to investigate the optimal treatment dose for further liver supportive treatment experiments during e.g. hepatic ischemia-reperfusion injury, toxic liver injury or liver inflammation.

Beside the anti-bacterial effect, taurolidine reduced the pro-inflammatory cytokines such as TNF alpha, IL-6 and IL-8 [7–13] and led to endotoxin inactivation [3]. Since the publication of Arlt et al. reporting severe liver toxicity after long-term oncological treatment of osteosarcoma in rodents with high doses of taurolidine [30], no severe toxic side effects had been reported [28, 29]. But Arlt et al. showed in mice that taurolidine led to hepatotoxicity with histological and morphological changes as well as alterations of serum liver enzymes after 25 days of a daily high-dose intraperitoneal treatment, whereas a low-dose treatment showed no hepatotoxicity [30]. These reported results are similar to our results after short-term treatment with taurolidine in rats and in the in vitro models, whereas the used doses in our in vivo study were about three times lower than in the previous study. Altogether it is difficult to compare exactly effects of the drug dosages between the different in vitro models and animal studies. So far, there are only few case reports of systemical use of taurolidine in humans available [46–48]. In these reports, patients were treated intravenously with taurolidine with a dose up to 300 mg/kg body weight, for glioblastoma, gastric cancer or metastatic melanoma [46–48]. In these few patients, no severe drug toxicity was seen, but in one patients elevated liver enzymes were reported [48]. In addition, there are several studies in which taurolidine was applied locally to the peritoneal or pleural cavity and for catheter-lock treatment, respectively [1, 4, 15, 16]. The toxic concentration of taurolidine identified in this study, was consequently lower than the used concentration in previous clinical case studies, but the toxic concentration in human subjects remains difficult to extrapolate. Finally, this is the first study investigating and comparing the effects of taurolidine in a liver-on-a-chip model, cell cultures and an animal in vivo model. Therefore, the exact comparison and interpretation of each system will be an issue in further investigations.

In this study we used three different experimental models with similar results reflecting robust and reliable findings. In contrast to monoculture experiments, the use of a microfluidically supported liver biochip comprising human cells provides an opportunity to investigate liver processes in a standardized manner [35, 49]. Because of direct cell-to-cell interactions of different hepatic cells within a microfluidic setting, liver biochips represent a more physiologic condition, but beside of macrophages other immune cells are missing in the model.

Even though the exact mechanism and responsible effector cells were not investigated, the main negative effect of taurolidine might be a direct toxic effect on hepatocytes, as the treatment even led to hepatic cell death in hepatocyte monocultures. This effect is emphasized by the increase and changes of serum enzymes reflecting hepatic cell death and necrosis. Even though the parameter values were in the maximum only doubled between the treatment groups, we are convinced that these changes are still relevant in this short-term investigation.

The increase of IL-6 in the liver biochip might be a response of Kupffer cells on the induced hepatic injury and inflammation. As other immune cells (NK cells, T- and B-cells) are lacking in the liver biochip model, pro- and anti-inflammatory pathways and processes between these cells are inexistent and might lead to an overwhelming inflammatory reaction. The effect of increased IL-6 was not seen in rats which is probably due to the lower taurolidine doses. In addition, as taurolidine has an anti-inflammatory effect during acute inflammation in vivo, taurolidine might lead to a protection against hepatoxicity and cytokine release in the in vivo model.

The lack of histological hepatic alteration in the in vivo model might be further explained by the fact that the observation period of 24 h was quite short. Hepatic necrosis is often seen not before 48 to 72 h after induction of liver injury.

A major experimental limitation should be noted: commercially available injectable taurolidine solution used in this study has a concentration of 2%. Therefore, only a restricted volume and concentration of taurolidine could be injected into rats. Thus, it was impossible to reach high hepatotoxic concentrations in rats as those used in in vitro experiments.

As the main toxic effect is dose dependent, further investigations of a potential protective effect of taurolidine in liver injury and inflammation, this toxic threshold value should be taken into account to avoid additional direct hepatotoxic drug effects.

## Conclusions

In conclusion, short-term treatment with taurolidine is associated with dose-dependent toxic liver injury with decreased hepatocyte survival in in vitro cell culture experiments and in a novel microfluidic hepatic biochip. In addition, liver dysfunction measured by serum levels of liver enzymes in rats showed direct toxic effects on the liver with high-dose treatment.

## Additional file

Additional file 1: Figure S1. Cell survival and LC50 of HepaRG cells after tauroldine treatment for 24 h was assessed by WST-assay. The LC50 for taurolidine was about 125 μg/ml (*n* = 6 per concentration, error bars = standard deviation). (TIFF 169 kb)

## Abbreviation

ASAT: Aspartate aminotransferase
COC: Cyclic olefin copolymers
GLDH: Glutamate dehydrogenase
GM-CSF: Granulocyte macrophage colony-stimulating factor
H/E: Hematoxylin/eosin
HUVEC: Human umbilical vein endothelial cells
IL: Interleukin
NK: Natural killer
PBMC: Peripheral blood mononuclear cells
TNF alpha: Tumor necrosis factor alpha

## References

[1] Linder MM, Ott W, Wesch G: [Antibacterial therapy of purulent peritonitis: a prospective randomized study on the effects of antibiotics and taurolin, a new chemotherapeutic and antiendotoxic agent (author’s transl)]. Chir Forum Exp Klin Forsch. 1980:67–71. 7389482

[2] Staubach KH (1997). Adjuvant therapy of peritonitis with taurolidine. Modulation of mediator liberation. Langenbecks Arch Chir.

[3] Reith HB (1997). Therapy of peritonitis today. Surgical management and adjuvant therapy strategies. Langenbecks Arch Chir.

[4] Conlan AA, Abramor E, Delikaris P, Hurwitz SS (1983). Taurolidine instillation as therapy for empyema thoracis. A prospective study of 50 patient. S Afr Med J.

[5] Neary PM, Hallihan P, Wang JH, Pfirrmann RW, Bouchier-Hayes DJ, Redmond HP (2010). The evolving role of taurolidine in cancer therapy. Ann Surg Oncol.

[6] Erb F, Febvay N, Imbenotte M (1982). Structural investigation of a new organic antiseptic: Taurolidine A spectroscopic study of its stability and equilibria in various solvents. Talanta.

[7] Traub WH, Leonhard B, Bauer D (1993). Taurolidine: in vitro activity against multiple-antibiotic-resistant, nosocomially significant clinical isolates of Staphylococcus aureus, Enterococcus faecium, and diverse Enterobacteriaceae. Chemotherapy.

[8] Sendt W, Mansouri E, Schmitt-Graeff A, Wolff-Vorbeck G, Schoffel U (2002). Influence of antiseptic agents on interleukin-8 release and transmigration of polymorphonuclear neutrophils in a human in vitro model of peritonitis. Surg Infect.

[9] Rosman C, Westerveld GJ, van Oeveren W, Kooi K, Bleichrodt RP (1996). Effect of intraperitoneal antimicrobials on the concentration of bacteria, endotoxin, and tumor necrosis factor in abdominal fluid and plasma in rats. Eur Surg Res.

[10] Rosman C, Westerveld GJ, Kooi K, Bleichrodt RP (1999). Local treatment of generalised peritonitis in rats; effects on bacteria, endotoxin and mortality. Eur J Surg.

[11] Watson RW, Redmond HP, Mc Carthy J, Bouchier-Hayes D (1995). Taurolidine, an antilipopolysaccharide agent, has immunoregulatory properties that are mediated by the amino acid taurine. J Leukoc Biol.

[12] Marcinkiewicz J, Kurnyta M, Biedron R, Bobek M, Kontny E, Maslinski W (2006). Anti-inflammatory effects of taurine derivatives (taurine chloramine, taurine bromamine, and taurolidine) are mediated by different mechanisms. Adv Exp Med Biol.

[13] Leithauser ML, Rob PM, Sack K (1997). Pentoxifylline, cyclosporine A and taurolidine inhibit endotoxin-stimulated tumor necrosis factor-alpha production in rat mesangial cell cultures. Exp Nephrol.

[14] Cullis PS, McKee RF (2011). Taurolidine lock - experience from the West of Scotland. Clin Nutr.

[15] Dumichen MJ, Seeger K, Lode HN, Kuhl JS, Ebell W, Degenhardt P, Singer M, Geffers C, Querfeld U (2012). Randomized controlled trial of taurolidine citrate versus heparin as catheter lock solution in paediatric patients with haematological malignancies. J Hosp Infect.

[16] Fontsere N, Cardozo C, Donate J, Soriano A, Muros M, Pons M, Mensa J, Campistol JM, Navarro-Gonzalez JF, Maduell F (2014). Tunneled catheters with taurolidine-citrate-heparin lock solution significantly improve the inflammatory profile of hemodialysis patients. Antimicrob Agents Chemother.

[17] Haag GM, Berger AK, Jager D (2011). Treatment of long-term catheter-related bloodstream infections with a taurolidine block: a single cancer center experience. J Vasc Access.

[18] Hoksch B, Rufer B, Gazdhar A, Bilici M, Beshay M, Gugger M, Schmid RA (2009). Taurolidine in the prevention and therapy of lung metastases. Eur J Cardiothorac Surg.

[19] Aceto N, Bertino P, Barbone D, Tassi G, Manzo L, Porta C, Mutti L, Gaudino G (2009). Taurolidine and oxidative stress: a rationale for local treatment of mesothelioma. Eur Respir J.

[20] Braumann C, Jacobi CA, Rogalla S, Menenakos C, Fuehrer K, Trefzer U, Hofmann M (2007). The tumor suppressive reagent taurolidine inhibits growth of malignant melanoma--a mouse model. J Surg Res.

[21] Braumann C, Gutt CN, Scheele J, Menenakos C, Willems W, Mueller JM, Jacobi CA (2009). Taurolidine reduces the tumor stimulating cytokine interleukin-1beta in patients with resectable gastrointestinal cancer: a multicentre prospective randomized trial. World J Surg Oncol.

[22] Chromik AM, Daigeler A, Hilgert C, Bulut D, Geisler A, Liu V, Otte JM, Uhl W, Mittelkotter U (2007). Synergistic effects in apoptosis induction by taurolidine and TRAIL in HCT-15 colon carcinoma cells. J Invest Surg.

[23] Chromik AM, Daigeler A, Bulut D, Flier A, May C, Harati K, Roschinsky J, Sulberg D, Ritter PR, Mittelkotter U (2010). Comparative analysis of cell death induction by Taurolidine in different malignant human cancer cell lines. J Exp Clin Cancer Res.

[24] Darnowski JW, Goulette FA, Cousens LP, Chatterjee D, Calabresi P (2004). Mechanistic and antineoplastic evaluation of taurolidine in the DU145 model of human prostate cancer. Cancer Chemother Pharmacol.

[25] Karlisch C, Harati K, Chromik AM, Bulut D, Klein-Hitpass L, Goertz O, Hirsch T, Lehnhardt M, Uhl W, Daigeler A (2013). Effects of TRAIL and taurolidine on apoptosis and proliferation in human rhabdomyosarcoma, leiomyosarcoma and epithelioid cell sarcoma. Int J Oncol.

[26] Daigeler A, Brenzel C, Bulut D, Geisler A, Hilgert C, Lehnhardt M, Steinau HU, Flier A, Steinstraesser L, Klein-Hitpass L (2008). TRAIL and Taurolidine induce apoptosis and decrease proliferation in human fibrosarcoma. J Exp Clin Cancer Res.

[27] Daigeler A, Chromik AM, Geisler A, Bulut D, Hilgert C, Krieg A, Klein-Hitpass L, Lehnhardt M, Uhl W, Mittelkotter U (2008). Synergistic apoptotic effects of taurolidine and TRAIL on squamous carcinoma cells of the esophagus. Int J Oncol.

[28] Braumann C, Menenakos C, Atanassov V, Pfirrmann RW, Guenther N, Jacobi CA (2008). Leukopoiesis is not affected after intravenous treatment with the novel antineoplastic agent taurolidine. Results of an experimental study in rats. Eur Surg Res.

[29] Braumann C, Guenther N, Pohlenz J, Pfirrmann RW, Menenakos C (2009). Wound healing is not impaired in rats undergoing perioperative treatment with the antineoplastic agent taurolidine. Eur Surg Res.

[30] Arlt MJ, Walters DK, Banke IJ, Steinmann P, Puskas GJ, Bertz J, Rentsch KM, Ehrensperger F, Born W, Fuchs B (2012). The antineoplastic antibiotic taurolidine promotes lung and liver metastasis in two syngeneic osteosarcoma mouse models and exhibits severe liver toxicity. Int J Cancer.

[31] Arlt MJ, Born W, Fuchs B (2012). Taurolidine: Mode of administration in mouse tumor models. Int J Cancer.

[32] Gripon P, Rumin S, Urban S, Le Seyec J, Glaise D, Cannie I, Guyomard C, Lucas J, Trepo C, Guguen-Guillouzo C (2002). Infection of a human hepatoma cell line by hepatitis B virus. Proc Natl Acad Sci U S A.

[33] Wallert M, Mosig S, Rennert K, Funke H, Ristow M, Pellegrino RM, Cruciani G, Galli F, Lorkowski S, Birringer M (2014). Long-chain metabolites of alpha-tocopherol occur in human serum and inhibit macrophage foam cell formation in vitro. Free Radic Biol Med.

[34] Mosig S, Rennert K, Krause S, Kzhyshkowska J, Neunubel K, Heller R, Funke H (2009). Different functions of monocyte subsets in familial hypercholesterolemia: potential function of CD14+ CD16+ monocytes in detoxification of oxidized LDL. FASEB J.

[35] Raasch M, Rennert K, Jahn T, Peters S, Henkel T, Huber O, Schulz I, Becker H, Lorkowski S, Funke H (2015). Microfluidically supported biochip design for culture of endothelial cell layers with improved perfusion conditions. Biofabrication.

[36] Beldi G, Wu Y, Sun X, Imai M, Enjyoji K, Csizmadia E, Candinas D, Erb L, Robson SC (2008). Regulated catalysis of extracellular nucleotides by vascular CD39/ENTPD1 is required for liver regeneration. Gastroenterology.

[37] Fahrner R, Patsenker E, de Gottardi A, Stickel F, Montani M, Stroka D, Candinas D, Beldi G (2014). Elevated liver regeneration in response to pharmacological reduction of elevated portal venous pressure by terlipressin after partial hepatectomy. Transplantation.

[38] Fahrner R, Trochsler M, Corazza N, Graubardt N, Keogh A, Candinas D, Brunner T, Stroka D, Beldi G (2014). Tumor necrosis factor-related apoptosis-inducing ligand on NK cells protects from hepatic ischemia-reperfusion injury. Transplantation.

[39] Jambekar AA, Palma E, Nicolosi L, Rasola A, Petronilli V, Chiara F, Bernardi P, Needleman R, Brusilow WS (2011). A glutamine synthetase inhibitor increases survival and decreases cytokine response in a mouse model of acute liver failure. Liver Int.

[40] Wang HX, Liu M, Weng SY, Li JJ, Xie C, He HL, Guan W, Yuan YS, Gao J (2012). Immune mechanisms of Concanavalin A model of autoimmune hepatitis. World J Gastroenterol.

[41] Brenner C, Galluzzi L, Kepp O, Kroemer G (2013). Decoding cell death signals in liver inflammation. J Hepatol.

[42] Zhai Y, Busuttil RW, Kupiec-Weglinski JW (2011). Liver ischemia and reperfusion injury: new insights into mechanisms of innate-adaptive immune-mediated tissue inflammation. Am J Transplant.

[43] Tsutsui H, Nishiguchi S (2014). Importance of Kupffer cells in the development of acute liver injuries in mice. Int J Mol Sci.

[44] Zider AD, Zopey R, Garg R, Wang X, Wang TS, Deng JC. Prognostic significance of infections in critically ill adult patients with acute liver injury: a retrospective cohort study. Liver Int. 2016;36(8):1143–50. 10.1111/liv.13073 PMC6023407 26801954

[45] Karvellas CJ, Pink F, McPhail M, Austin M, Auzinger G, Bernal W, Sizer E, Kutsogiannis DJ, Eltringham I, Wendon JA (2010). Bacteremia, acute physiology and chronic health evaluation II and modified end stage liver disease are independent predictors of mortality in critically ill nontransplanted patients with acute on chronic liver failure. Crit Care Med.

[46] Stendel R, Picht T, Schilling A, Heidenreich J, Loddenkemper C, Janisch W, Brock M (2004). Treatment of glioblastoma with intravenous taurolidine. First clinical experience. Anticancer Res.

[47] O'Brien GC, Cahill RA, Bouchier-Hayes DJ, Redmond HP (2006). Co-immunotherapy with interleukin-2 and taurolidine for progressive metastatic melanoma. Ir J Med Sci.

[48] Braumann C, Winkler G, Rogalla P, Menenakos C, Jacobi CA (2006). Prevention of disease progression in a patient with a gastric cancer-re-recurrence. Outcome after intravenous treatment with the novel antineoplastic agent taurolidine. Report of a case. World J Surg Oncol.

[49] Rennert K, Steinborn S, Groger M, Ungerbock B, Jank AM, Ehgartner J, Nietzsche S, Dinger J, Kiehntopf M, Funke H (2015). A microfluidically perfused three dimensional human liver model. Biomaterials.

