# Supplementary figures and images for: Short-term treatment with taurolidine is associated with liver injury
Source: BMC Pharmacol Toxicol. 2017 Aug 11;18:61. doi: 10.1186/s40360-017-0168-z (PMC5553585; doi:10.1186/s40360-017-0168-z)

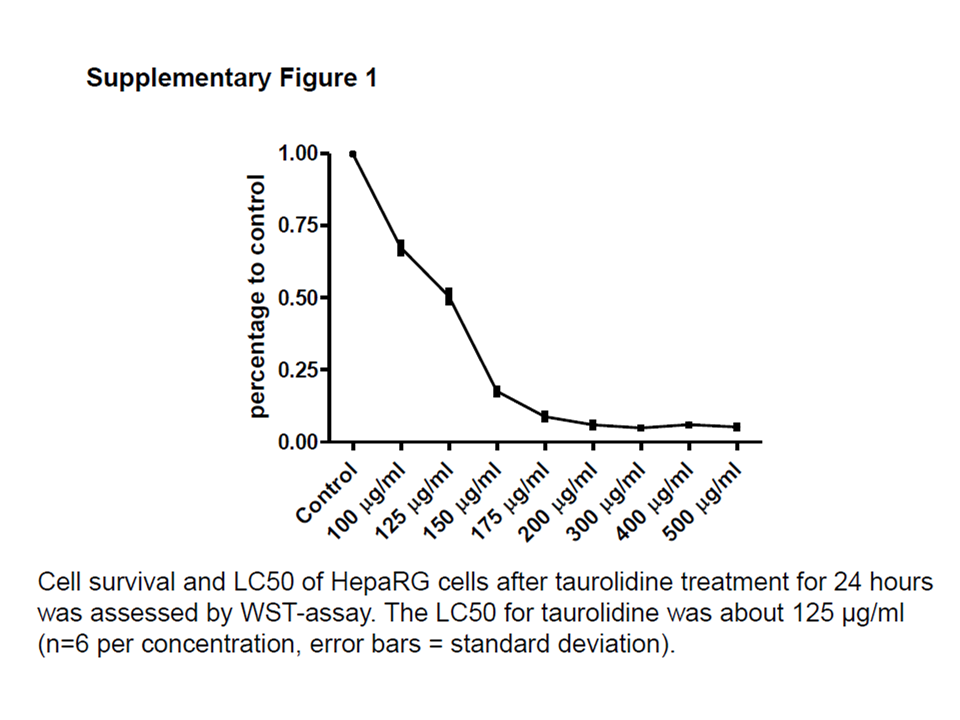

Supplement: Additional file 1: Figure S1. — Cell survival and LC50 of HepaRG cells after tauroldine treatment for 24 h was assessed by WST-assay. The LC50 for taurolidine was about 125 μg/ml (n = 6 per concentration, error bars = standard deviation). (TIFF 169 kb) [file 40360_2017_168_MOESM1_ESM.tif]
